# Hunger modulates perceptions of food health but not taste in restricted eaters

**DOI:** 10.3389/fpsyg.2023.1212778

**Published:** 2023-07-06

**Authors:** Lucia Herrero, Cindy E. McCrea

**Affiliations:** Social Determinants of Cognition, Behavior, and Health Lab, Department of Psychological Science, Boise State University, Boise, ID, United States

**Keywords:** hunger, eating behaviors, restricted eating, health perception, taste perception, food choice

## Abstract

**Introduction:**

Food taste and health perceptions are of particular interest for their implications on food choice. Most *in vivo* food choice studies experimentally control for hunger via a set preload or fast.

**Methods:**

To explore how hunger may interact with these perceptions to impact food decisions, we compared taste and health perceptions of sweet and savory obesogenic food items among hungry or satiated participants with varying restrained eating tendencies.

**Results:**

In our sample of 232 adults (*M* BMI = 25.9; *M* age = 36.4 yrs), highly palatable foods were perceived as tasty but unhealthy. Tastiness ratings were high, consistent across restrained eating groups, and unassociated with hunger. Perceptions of health, however, were impacted by the interaction of restrained eating group and hunger. Amongst hungry participants only, a graded association between restrained eating group and perceptions of health emerged for both food types. Specifically, hungry and highly restrained eaters viewed sweet foods as 2.8x healthier and savory foods as 2.1x healthier than their satiated counterparts.

**Discussion:**

Our data suggest that hunger predicts differential health perceptions, but not tastiness ratings, among restrained eaters. We argue that the generalization of food perception data–especially among different eater types–may be limited if the continuum of hunger level is experimentally constrained. Therefore, hunger is a critical dynamic to consider in explorations of food perceptions and eating behavior in restrained eaters.

## 1. Introduction

In Western cultures rife with highly palatable but obesogenic foods, an increasing number of individuals attempt to prevent obesity by restricting their caloric intake (Hofmann et al., 2014; Stierman et al., 2020). However, these intentions to moderate what, when, and how much is consumed are not always successful (Mann et al., 2007; Reichenberger et al., 2020). A common explanation for this regulatory failure is related to the hedonic nature of food; for restricted eaters, some foods may seem too tempting to resist (Papies et al., 2007). Nevertheless, in clinically-restricted eaters, calorically-dense foods may evoke aversion (Foerde et al., 2020). For this reason, perceptions of food taste and health are of particular interest due to their implications for food choice. Food choice is a complex phenomenon influenced by biological, psychological, demographic, and situational factors (Zandstra et al., 2001; Stok et al., 2017; Kowalkowska et al., 2018; Foerde et al., 2020; Londerée and Wagner, 2021). Determinants of food choice include stress, nutritional value, “convenience, sensory appeal, natural content, ethical concern, weight control, mood, familiarity” (Marty et al., 2021, para. 1).

Neural representations of the taste and healthiness of foods are automatically encoded when we encounter food cues (Londerée and Wagner, 2021) and affect neural processes related to self-control (Hare et al., 2009), reward (King et al., 2018), and valuation of food (Hare et al., 2009). The impact of each valence on food choice may differ for those with varying clinical eating patterns (Steinglass et al., 2015; Berner et al., 2017; Stojek et al., 2018; Foerde et al., 2020). For example, while anorexia nervosa patients base food decisions primarily on the perceived healthiness of foods, both dieting and non-dieting healthy participants’ choices are driven by taste (Foerde et al., 2020). These differences may be the result of learned food avoidance in clinically restrained eaters via a fear conditioning paradigm (Garcia-Burgos et al., 2023; Simonazzi et al., 2023). However, when healthy participants are hungry, the impact of taste on eating behavior is diminished in favor of food availability and portion size (Hoefling and Strack, 2010). Previous studies characterize how disordered eating influences perception of food stimuli. In this study, we seek to extend this field of research by presenting data on food perception among individuals with the nonclinical eating tendency of restrained eating in both the hungry and satiated state.

Restrained eaters are categorized as those who “frequently or regularly try to control their weight by limiting their caloric intake” (Hofmann et al., 2014, para. 1). Ironically, simultaneously, restrained eaters tend to experience stronger urges to consume food and overeat more often than non-restrained eaters (Herman and Polivy, 1975, 2022). Herman and Polivy (1975)’s boundary model posits that due to the cyclical pattern of dieting and overeating, restrained eaters experience lower hunger and higher satiety thresholds compared to unrestrained eaters. Palatable food cues lead restrained eaters to envision the enjoyment associated with consumption significantly more than non-restrained eaters (Papies et al., 2007), and these hedonistic thoughts make the dieting goal cognitively harder to access (Stroebe, 2008). Nevertheless, the tempting nature of food can be mitigated through appropriate cognitive signals: an intervention aimed at priming restrained participants to remember dieting goals prior to food cue exposure was successful at curtailing overeating (Papies and Hamstra, 2010).

Thus, for restrained eaters, self-regulation failure may be the result of goal conflict: the goal of hedonic enjoyment of food may temporarily inhibit dieting goals (Papies and Hamstra, 2010). However, ratings of food item tastiness, an inherently hedonic aspect of eating, were not different between restrained and unrestrained eaters in the limited work reporting this outcome (Roefs et al., 2005). Indeed, others have demonstrated that while restrained eaters do not display immediate implicit bias against palatable foods, reflexive measures which allow for value-based judgments are more negative (Hoefling and Strack, 2008). Additionally, dieters more accurately estimate the caloric content of highly palatable foods than non-dieters, are more attuned to their negative properties, and rate them less positively overall, perhaps because they are incongruent with dieting goals (Carels et al., 2007; Papies and Hamstra, 2010; Papies, 2013). Therefore, it is unclear whether restrained eaters differ from non-restrained eaters in their base evaluation of food tastiness and health or if other factors such as environmental cues and internal states such as hunger account for behavioral differences in this population.

The goal of this protocol was to investigate the possible impact of restrained eating tendencies on the health and taste perceptions of common palatable food items using the Food Folio (Lloyd et al., 2020), a recent comprehensive portfolio of food images with large-scale, normative data collected via an online Amazon Mechanical Turk sample. We further sought to explore the impact of hunger on these ratings. In accordance with the literature, we expected that restricted eaters would rate obesogenic foods as less healthy than unrestricted eaters. We independently hypothesized that hunger would enhance the tastiness ratings of food items, especially in restricted eaters who are particularly susceptible to the hedonic nature of food.

## 2. Methods

### 2.1. Participants and procedures

Our sampling goal for this project was 200 adults from the United States. Existing work evaluating perceptions of tastiness and health in restricted eaters relied on samples of 110, 69, and 56 (Roefs et al., 2005; Foerde et al., 2020). The author team judged that this larger size would be necessary to elucidate an interaction with hunger. To collect data from a broader context, we recruited participants via MTurk, a crowdsourcing marketplace operated by Amazon. MTurk is an ecologically valid and reliable recruitment tool with no significant difference in attention and buy-in compared to in-person participants (Thomas and Clifford, 2017). On the MTurk platform, workers viewed the title, a brief description, estimated time, and the reward for participation in the study. On the next page, the cover letter specified that participants were required to be 18 years of age, proficient in English, and have access to a computer or laptop with a physical keyboard. To proceed, participants provided informed consent, then accessed the survey hosted via Qualtrics. Workers were compensated with a $5 USD Amazon electronic gift card for completing 30–40 min of work based on the US standard minimum wage based on MTurk best practices (Aguinis et al., 2021).

We collected 383 surveys via Amazon MTurk. We removed 81 incomplete responses, 33 repeats by the same participant (only first use was retained), 1 for a nonsensical answer in a text field, 12 due to food restrictions that would interfere with the food choice task (i.e., dairy), 22 from Workers who took the survey from outside the US, and 2 for durations over 5,000 *s*. The final sample comprised 232 participants, including 138 men and 77 women (*n* = 17 failed to report sex). The mean age of the sample was 36.4 years (10.9 *SD*) and mean BMI was 25.9 (5.7 *SD*).

### 2.2. Measures

#### 2.2.1. Eating behavior questions

Respondents completed a number of eating-related questions. The hunger score variable was calculated as the mean score from two questions measured on a slider scale from 0 to 100: how hungry are you right now, and how strong is your desire to eat right now? We used a median split to categorize hungry and satiated participants so that scores above 50 represented hunger.

#### 2.2.2. Dutch eating behaviors questionnaire

We included five items (questions 1, 2, 5–7) from the restrained eating subset of the Dutch Eating Behaviors Questionnaire (van Strien et al., 1986). These questions specifically measured participants’ tendency to eat less to mitigate overconsumption and weight gain. The reliability and validity of this tool are well-established; DEBQ-restricted eating scores correlate well with another measure of restriction (Van Strien et al., 1986). The restraint scale has high test–retest reliability (*r = 0.*92) (Allison et al., 1992), and high internal consistency (*α* = 95) (van Strien et al., 1986). Scores were indicated on a sliding scale ranging from 0 to 100. A restrained eating score was calculated as the average of all restrained eating questions. Participants were grouped into the “high” restraint category with a score above 61, “average” between 30 and 60.9, and “low” below 30.

#### 2.2.3. Food rating task

In this study, we were specifically interested in evaluating perceptions of obesogenic foods in restrained eaters due to the hedonic nature of tempting foods. Participants rated the perceived health and taste of six obesogenic food items disguised among 17 filler items, which included a variety of additional foods, such as healthy and unhealthy, unprocessed and processed, sweet and savory, single item and combo items (e.g., veggies with ranch dipping sauce), and snacks and meals to appeal to a wide range of palates. The six obesogenic foods included three savory items (pizza, fries, chicken nuggets) and three sweet items (brownie, donut, ice cream), which were selected because of their very high tastiness but very low health ratings in the original Food Folio study (Lloyd et al., 2020).

All images utilized were sourced from the Food Folio by Columbia Center for Eating Disorders: A Freely Available Food Image Database (Lloyd et al., 2020). Factor analysis of ratings of Food Folio images indicates that ratings are reliable and valid. Participants were instructed to only consider the health or taste of the food in their rating and not consider any other factors. Response options were on a sliding scale ranging from *not at all tasty/healthy* (0) to *neutral* (5) to *very tasty/healthy* (10). The food item images were randomized within each block so that the participants did not rate the foods in the same order.

#### 2.2.4. Demographic questions

Finally, we included demographic questions to account for potential differences in sex, age, and body mass index (BMI) (see Table 1).

**Table 1:** Sample demographics by restrained eating group.

Restricted eating category	High (*n* = 98; 34% Female)		Average (*n* = 79; 30% Female)		Low (*n* = 55; 54% Female)	
	*M*	*SD*	*M*	*SD*	*M*	*SD*
Age	34.9	9.5	35.6	11.8	39.9	11.6
BMI	26.2	6.3	25.1	5.2	26.6	5.5
Restrained eating score	76.6	10.2	48.2	8.7	11.3	10.2

#### 2.2.5. Statistical analysis

We used the PROC MIXED procedure in SAS 9.4 to test the fixed effects of restrained eating tendencies (high, average, low), and hunger (high, low) on the average ratings of taste and health for the three sweet obesogenic items and the three savory obesogenic foods. Although PROC MIXED allows the inclusion of random and repeated effects, none were included in these models. The base models included sex and BMI as covariates, as restricted eating tends to be more prevalent among women and is correlated with weight history (Cachelin and Regan, 2006). Model selection was based on optimizing fit statistics (evaluated as lowest Bayesian Information Criterion), both covariates were retained in the final model because they enhanced model fit. *Post hoc* tests were adjusted for multiple comparisons using the Tukey procedure. Least-squares means, standard deviations in descriptions and tables, and standard errors in figures for visual clarity are reported. PROC GLM two-way ANOVA analysis was conducted to produce Type III SS effect size estimates.

## 3. Results

As expected, participants generally viewed the six high-calorie food items as tasty (*M* = 8.045 out of 10, *SD* = 1.179) and not very healthy (*M* = 3.831 out of 10, *SD* = 2.600). Thirty-four percent of the sample were categorized as highly restrained eaters, another 42% as average, and 24% reported low levels of restrained eating behaviors. Just over half (52%) of respondents fell into the hunger category. An overview of the sample characteristics by restrained eating group is provided in Table 1.

**Figure 1:**
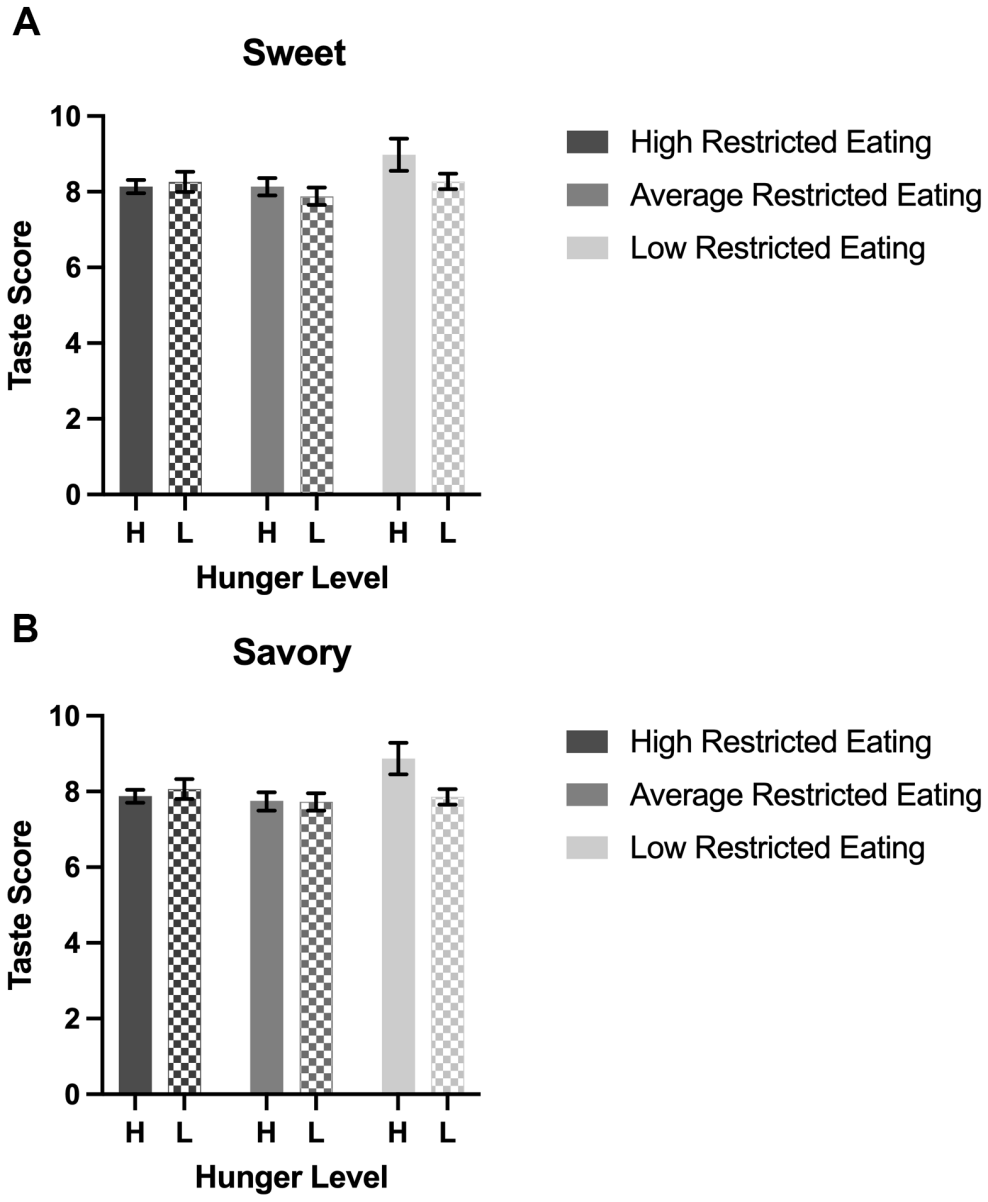
Perceived taste of obesogenic items by hunger level and restricted eating groups. Error bars indicate standard error. **(A)** Average taste ratings of the three sweet items categorized by the interaction of restricted eating groups and high (H) vs. low (L) hunger. **(B)** Average taste ratings of the three savory items categorized by the interaction of restricted eating groups and high (H) vs. low (L) hunger.

**Figure 2:**
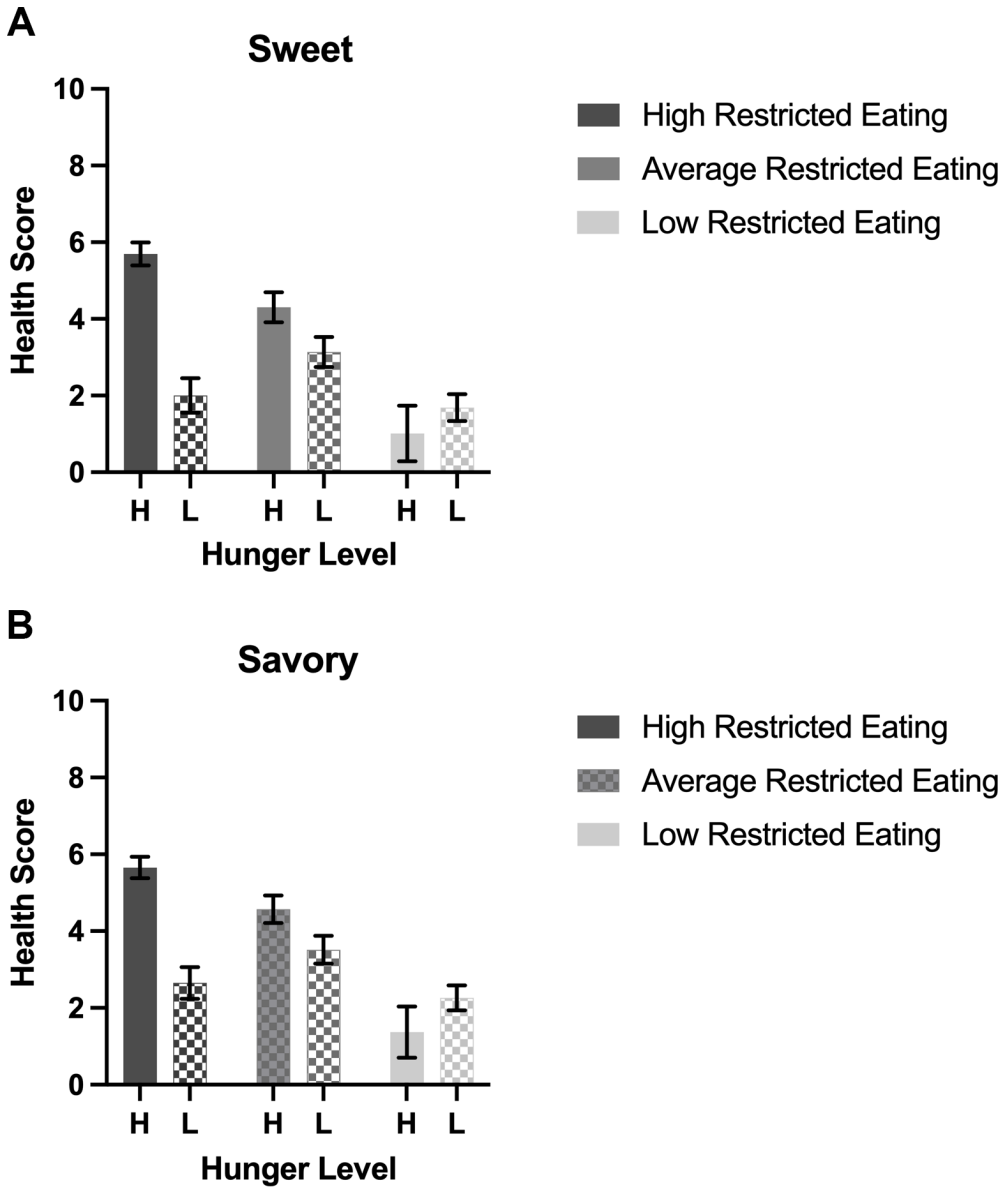
Perceived health of obesogenic items by restricted eating groups. Error bars indicate standard error. **(A)** Average health ratings of the three sweet items categorized by the interaction of restricted eating groups and high (H) vs. low (L) hunger. **(B)** Average health ratings of the three savory items categorized by the interaction of restricted eating groups and high (H) vs. low (L) hunger.

Participants perceived the sweet and savory items as similarly tasty regardless of hunger or restrained eating tendencies. Neither sex nor BMI predicted taste ratings (Figures 1A,B). The expected taste-enhancing effect of hunger was not present in any groups of eaters. We expected to find that restricted eaters would rate obesogenic foods as less healthy than their unrestricted counterparts. However, health perceptions of the savory obesogenic foods were predicted by the interaction of restrained eating category and hunger level (*p* < 0.0001; Figures 2A,B). Among the hungry participants, a graded association appears to be visible between restrained eating status and health perceptions. Specifically, among hungry participants, both high and average restrained eaters viewed savory palatable foods as healthier than those with low restrained eating scores (*Tukey p*s < 0.001), although the difference between the high and average restraint groups was not significant (*Tukey p* = 0.14). In satiated participants, there was no association between restrained eating status and health ratings of savory foods. Additionally, among the highly restrained eaters only, hunger predicted a large difference in health perceptions. Hungry and highly restrained eaters rated savory, palatable foods 2.1 times healthier than their satiated but also highly restrained counterparts (*Tukey p* < 0.0001); This effect of hunger was not present in the average or low restrained eaters (*Tukey p*s = 0.29 and 0.83, respectively). The effect size for the interaction of hunger and restricted eating category on the healthiness ratings of savory items was moderate (partial η^2^ = 0.0924). The covariate BMI, but not sex, also emerged as a significant independent predictor of health ratings in savory items (*p* = 0.04).

A very similar effect emerged for health ratings of sweet items (Figure 2A). Among the hungry participants, restrained eating status was associated with health perceptions in a graded manner that was statistically significant at all three levels (H vs. A restrained eaters *Tukey p* = 0.049; A vs. L *Tukey p* = 0.001). There was no association between restrained eating status and health ratings of sweet foods in satiated participants. Additionally, among the high restrained eaters only, hunger predicted a substantial difference in health perceptions: hungry and highly restrained eaters rated sweet, palatable foods 2.8 times healthier than their satiated but also highly restrained counterparts (*Tukey p* < 0.0001); This effect was not present in the average or low restrained eaters (*Tukey ps* = 0.27 and 0.96 respectively). The effect size for the interaction of hunger and restricted eating category on the healthiness ratings of sweet items was moderate (partial η^2^ = 0.1022). The covariate BMI, but not sex, also emerged as a significant independent predictor of health ratings in savory items (*p* = 0.02).

## 4. Discussion

Food taste is understood to drive food choices among healthy eaters while health perceptions drive choice in those with anorexia nervosa (Steinglass et al., 2015; Foerde et al., 2020). However, hunger may interact with these perceptions to impact food decisions. We compared perceptions of food taste and health for various palatable but obesogenic food items among those with varying restrained eating statuses who were hungry or satiated.

On average, highly palatable foods were perceived as tasty but unhealthy. Tastiness ratings were relatively high, and contrary to our hypothesis, were consistent across restrained eating groups despite hunger levels. This finding supports work by Roefs et al. (2005) who also found that tastiness ratings were not different for restrained and unrestrained eaters, and Hoefling and Strack (2008) who found no differences in immediate likability ratings of foods among eater types. Perceptions of healthiness, however, were impacted by the interaction of restrained eating group and hunger in our sample, but not in the hypothesized direction. Amongst hungry participants only, a graded association between restrained eating category and perceptions of health was evident for both food types; thus, higher restrained eating status was associated with higher health ratings. Congruently, hungry and highly restrained eaters rated both the sweet and savory obesogenic foods as healthier than their satiated but also highly restrained counterparts. Together these data suggest that although restrained eaters’ perceptions of food may not differ much from unrestrained eaters when satiated, hunger may render restrained eaters more vulnerable to overestimations of the health value–but not taste–of palatable foods.

Hunger, by definition, elevates the desirability of food. Fasting is known to modulate neural responses in reward-related areas (Uher et al., 2006; Führer et al., 2008; Siep et al., 2009). Siep et al. (2009) found evidence suggesting that the right insula, often associated with cue-induced drug cravings, was modulated by the interaction of high-calorie food cues and hunger. Siep et al. (2009) posit that this activation represents an increase in reward value of energy-dense food in the context of hunger and that hunger triggers an evolutionary urge to sequester high-calorie food to stave off starvation via optimal foraging theory. This is consistent with intuitive eating theories which emphasize how interoceptive hunger and satiety cues guide adaptive eating behavior (Herbert et al., 2013). Thus, while the hedonic aspect of food is always present, hunger in particular activates the salience of food-related stimuli. In our protocol, however, hunger did not affect the health valuations of all participants equally. Restrained eaters specifically overestimated food health when hungry. This is somewhat surprising given that restrained eaters are on average more accurate in their health valuations of food than non-restrained eaters (Carels et al., 2007). However, a longitudinal dieting study may have captured evidence that restricted eating changes the relationship between hunger and behavior; though unrelated before caloric restriction began, after 6 months of restrained eating, hunger score was significantly associated with giving in to cravings in a sample of overweight women (Gilhooly et al., 2007). Three theoretically-driven explanations for this interaction are discussed below.

It is well established that restrained eaters exhibit less self-regulatory control over eating behavior than unrestrained eaters (Herman and Mack, 1975; Herman and Polivy, 1975; Boon et al., 2002). Herman and Polivy’s (1975) boundary model posits that restrained eaters have lower hunger and higher satiety thresholds due to chronic dieting, suggesting the possibility that restricted eaters feel hunger more intensely and frequently than unrestricted eaters. It follows that hunger may be an urgent state that transgresses the diet boundary, resulting in skewed perceptions of food valuation. Research in adjacent areas, including economics psychology, indicates that fasting individuals take greater risk compared to satiated individuals, driven by a desire to reach a metabolic reference point (Symmonds et al., 2010). For restrained eaters, hunger may more quickly approach a condition similar to fasting, evoking a willingness to risk the diet–thereby transgressing the diet boundary–in order to satisfy the urge for metabolic homeostasis.

Wegner’s Ironic Process Theory (1994) suggests an alternative possible explanation for this effect. The theory is based on the idea that self-control relies on both a monitoring process that detects offensive stimuli and an operating process that works to avoid them. The latter is more sensitive to distraction. In the reduced capacity state, the individual retains sensitivity to the mental content they are actively trying to avoid but not the processing to avoid it (Wegner, 1994). Thus, ironically, they are more likely to identify and consume foods they intended to avoid. In this view, our satiated restricted eaters may have assessed food health with respect to the operating goal of avoiding calorically-dense foods. In the context of hunger, these restrained eaters’ goal-oriented operating process is diminished but their monitoring process actively draws attention to high-calorie foods. This increased awareness of palatable foods specific to restrained eaters coupled with a hunger-induced drive toward high-calorie options may explain why restrained eaters were the only participants vulnerable to overestimations of health.

Alternatively, Papies and Hamstra (2010) suggest that restricted eaters are subject to a hedonic orientation towards food, which can override dieting goals when spontaneously activated. Indeed, the incongruence between restrained eaters’ explicit and implicit attitudes toward food suggests that internal struggle is present (Hoefling and Strack, 2008). Our data may offer an additional nuance to this goal conflict paradigm: hunger may exacerbate the desire to enjoy palatable foods enough to induce a state of cognitive dissonance, or intense internal struggle around the conflicting goals. A host of studies demonstrate that when faced with a direct conflict between our desired or actual behavior and what we believe to be good, individuals are likely to change the latter to justify the former (Festinger, 1957). This is understood to be an emotion regulation response, reducing the unpleasant emotions associated with dissonance (Cancino-Montecinos et al., 2018). The Food Cognitive Dissonance Model was recently developed to guide diet-related dissonance research (Ong et al., 2017). A few limited studies evaluating the conflict between adoration of animals and meat-eating behavior have produced evidence supporting the view that cognitive dissonance can be applied to eating behaviors (Dowsett et al., 2018; Rothgerber, 2020). Additionally, evidence demonstrates that individuals will justify consuming unhealthy foods by endorsing convenient beliefs, particularly in the presence of tempting food cues (Hill et al., 2016). In the case of restrained eaters, hunger may enhance dissonance to such a degree that beliefs about the health of palatable foods are adjusted to align with desire unless otherwise primed (Papies and Hamstra, 2010; Hill et al., 2016). This theory may lend an explanation to the surprising differences in health perceptions, understood to be cognitions based on knowledge and therefore assumed to be less variable than other aspects of the eating experience (i.e., temptingness, craving strength). However, the view that internal conflict may induce perception changes is challenged by recent work suggesting that significant conscious conflict may not be present when restrained eaters make food choices (van der Laan et al., 2014; Georgii et al., 2020).

Our protocol, rather uniquely in this body of literature, evaluated the interaction between varying self-reported hunger levels and restricted eating tendencies on perceptions of food taste and health. The stark differences between health perceptions among the hungry and satiated restricted eaters signal the need for more work to evaluate how hunger may alter food-related perceptions and, potentially, eating behavior in this population. A full one-third of participants in our sample fell into the affected group, and although normative data is scarce, estimates suggest that 10%–20% of U.S. citizens may display restricted eating tendencies (Rand and Kuldau, 1991; Cachelin and Regan, 2006). This work is limited by the small number of obesogenic foods which were rated (n = 6). However, the hunger by restrained eating interaction was replicated consistently in the statistical models predicting the ratings of the three savory and the three sweet obesogenic food items. The consistency of the direction and magnitude of these effect lends credibility to the findings.

Notably, this data is cross-sectional and is therefore subject to the standard limitations of such designs. Further work is needed to confirm the effect observed here and experimental designs are needed to ascertain if hunger induces momentary changes in restricted eaters’ perceptions. These findings are also limited by the restrained eating assessment procedure. Just half of the DEBQ restrained eating questions were utilized. While strong factor loading values for these items (from van Strien et al., 1986) suggest they provide a reasonable estimate, utilizing the entire scale would strengthen these findings. Additionally, some data suggests that the Restraint Scale is a more valid measure of restrained eating behavior; future work should consider if classification using this alternate method produces similar results (Adams et al., 2019). Although relatively small compared to nationally representative samples, this sample is relatively large for this body of literature (Roefs et al., 2005; Foerde et al., 2020). However, our findings were based on data from a convenience sample of MTurk Workers. Therefore, our findings should be viewed more as hypothesis-generating rather than being representative of the general population.

In conclusion, our data suggest that hunger predicts differential health perceptions, but not tastiness ratings, among restrained eaters. Thus, hunger is a critical dynamic to consider in explorations of food perceptions and eating behavior in restrained eaters.

## Data availability statement

The raw data supporting the conclusions of this article will be made available by the authors, without undue reservation.

## Ethical statement

The studies involving human participants were reviewed and approved by Boise State University Office of Research Compliance Institutional Review Board. The patients/participants provided their written informed consent to participate in this study (Reference # 041-SB21-013).

## Author contributions

LH and CM contributed to the conceptualization and design of the study, conducted literature searches and summaries, and conducted statistical analysis. All authors contributed to the article and approved the submitted version.

## Conflict of interest

The authors declare that the research was conducted in the absence of any commercial or financial relationships that could be construed as a potential conflict of interest.

## Publisher’s note

All claims expressed in this article are solely those of the authors and do not necessarily represent those of their affiliated organizations, or those of the publisher, the editors and the reviewers. Any product that may be evaluated in this article, or claim that may be made by its manufacturer, is not guaranteed or endorsed by the publisher.
